# Gene profiling of the erythro- and megakaryoblastic leukaemias induced by the Graffi murine retrovirus

**DOI:** 10.1186/1755-8794-3-2

**Published:** 2010-01-26

**Authors:** Veronique Voisin, Philippe Legault, Diana Paulina Salazar Ospina, Yaacov Ben-David, Eric Rassart

**Affiliations:** 1 Laboratoire de Biologie Moléculaire, Département des Sciences Biologiques, Centre BioMed, Université du Québec à Montréal, Case Postale 8888 Succursale Centre-ville, Montréal, QC, H3C-3P8, Canada; 2 Sunnybrook Health Sciences Center, 2075 Bayview Ave. S223B, Toronto. ON, M4N 3M5, Canada

## Abstract

**Background:**

Acute erythro- and megakaryoblastic leukaemias are associated with very poor prognoses and the mechanism of blastic transformation is insufficiently elucidated. The murine Graffi leukaemia retrovirus induces erythro- and megakaryoblastic leukaemias when inoculated into NFS mice and represents a good model to study these leukaemias.

**Methods:**

To expand our understanding of genes specific to these leukaemias, we compared gene expression profiles, measured by microarray and RT-PCR, of all leukaemia types induced by this virus.

**Results:**

The transcriptome level changes, present between the different leukaemias, led to the identification of specific cancerous signatures. We reported numerous genes that may be potential oncogenes, may have a function related to erythropoiesis or megakaryopoiesis or have a poorly elucidated physiological role. The expression pattern of these genes has been further tested by RT-PCR in different samples, in a Friend erythroleukaemic model and in human leukaemic cell lines.

We also screened the megakaryoblastic leukaemias for viral integrations and identified genes targeted by these integrations and potentially implicated in the onset of the disease.

**Conclusions:**

Taken as a whole, the data obtained from this global gene profiling experiment have provided a detailed characterization of Graffi virus induced erythro- and megakaryoblastic leukaemias with many genes reported specific to the transcriptome of these leukaemias for the first time.

## Background

Human acute megakaryoblastic (FAB-AML7, [1]) and erythroleukaemias (FAB-AML6, [2]) are regarded as relatively rare entities of acute myeloid leukaemia but are associated with a very poor prognosis [3-7]. The poor outcome linked to these 2 types of leukaemias stems from a combination of failure to achieve complete remission, a high relapse rate and therapy-related toxicity, highlighting the need for more powerful therapies. Furthermore, AML6 or AML7 diagnosis represents a greater challenge than other types of acute myeloid leukaemia (AML) and additional markers are needed [8]. Furthermore, the blasts of patients with AML6 and AML7 share common markers [9] indicating that they originate from closely related haematopoietic lineages derived from a common bipotent progenitor [10,11].

We have recently shown that the murine retrovirus Graffi is able to induce a broad spectrum of leukaemias when inoculated into newborn mice. The leukaemias developed by these mice are of lymphoid (T-cell and B-cell) and non lymphoid (myeloid, erythroid and megakaryoblastic) origins. The incidence of erythro- and megakaryoblastic leukaemias is particularly high in NFS or FVB/n mice inoculated with the GV-1.4 variant of the Graffi virus [12]. The activation of the targeted proto-oncogene or the repression of tumor suppressor genes represents early events in the development of the murine leukaemia retrovirus (MuLV) induced leukaemia. It is then followed by a deregulation of numerous additional genes resulting in a cell, blocked at a very immature stage, which aggressively divides and escapes apoptosis. To analyze these cancerous signatures, we compared the gene profiles of each type of leukaemia (T-cell, B-cell, myeloid, erythroid, megakaryoblastic) induced by the Graffi virus. These analyses highlight many genes that may be potential oncogenes and may have a function related to erythropoiesis or megakaryopoiesis. The results support the importance of the known transcription factors *Gata1*, *Fog1*, *Fli1*, *Scl *and *Lmo2 *in both erythro- and megakaryoblastic leukaemias and the role of *Runx1*, *Pbx1*, *Meis*, *Evi1 *and *Evi3 *in the megakaryoblastic leukaemias. Moreover, numerous genes are being reported for the first time and some of these genes are candidate oncogenes: *Fgf3*, *Nmyc*, *Fap*, *Myct1*, *Gucy1a3*, *Gulp1 *and *Fkbp9 *specific to megakaryoblastic leukaemias and *Ssx2ip*, *Rab11a, Ncoa3*, *Snca*, *Ltbp2*, *Rabgef1 *and *Btbd14a *specific to erythroleukaemias. A screening for viral integrations was performed in mouse tumors. Several genes, amongst which *Kit*, *Gata2*, *Irf8 *and *Itga1*, were identified as potentially implicated in the onset development of the megakaryoblastic leukaemias.

## Methods

### Virus production and mice

GV-1.4 viral stock was made as previously described [12]. GV-1.4 viral particles (0.1 ml at a titer of 1.10^6 ^PFU/ml) were injected into 1 day newborn NFS mice. The mice were checked routinely for clinical signs of disease and moribund mice were sacrificed. Twenty-four diseased mice and 36 control mice were used for the microarray and RT-PCR experiments. Bone marrow cell suspension was prepared by flushing the femurs with IMDM 2% foetal bovine serum (FBS) and spleen cell suspension was prepared by mincing the spleen with scissors and aspirating the pieces up and down through a 1cc syringe in IMDM 2% FBS. The spleen and bone marrow cell suspensions were filtered through 70 μm cell strainers (Becton Dickson, Mississauga, Canada). All the experimental procedures are conformed to the Helsinki Declaration and were approved by the Animal Care and Use Committees of Université du Québec à Montréal.

### Flow cytometry analyses and cell sorting

The flow cytometry staining procedure was performed as previously described [12]. The antibodies used were as follows: CD4, CD8a, CD3, CD90, CD19, B220, CD11b, Gr1, CD71, Ter119, CD41, Kit and Sca1 (BD Pharmingen, Mississauga, Canada). The leukaemic populations were isolated from the haematopoietic organs by positive selection using magnetic beads with the EasySep Kit (StemCell Technologies, Vancouver, Canada) according to the manufacturer's protocol. The rates of purity and viability of the sorted cells were fixed to be equal to or greater than 95%. Leukaemic T-cells were sorted from the thymus of leukaemic NFS mice, B-cell from the enlarged lymph nodes and erythro- and megakaryoblastic leukaemic cells were sorted from the infiltrated spleen. Control cells were sorted from the haematopoietic organs of 12 pooled non-infected NFS mice: T-cells were obtained from the thymus, B-cells from the spleen, and erythroblasts from the bone marrow.

### RNA extraction and microarray processing

Total RNA was extracted from the sorted cell populations with Trizol reagent (Invitrogen, Burlington, Canada) followed by column purification using the Qiagen RNeasy Kit (QIAGEN, Mississauga, Canada) and processed for hybridization to Affymetrix GeneChip^® ^Mouse Genome 430 2.0 arrays (Genome Quebec Innovation Centre, Montreal, Canada).

### Data analysis

Data Set Normalization: Affymetrix MicroArray Suite version 5.0 was used to scan and quantify the arrays. Normalization of gene expression data were performed using the Bioconductor implementation of RMA (Robust Multi Array, B. Bolstad, University of California, Berkeley) available from the Flexarray software (1.2, R 2.7.2, [13]).

Unsupervised learning: Hierarchical clustering (complete linkage clustering, correlation uncentered, [14]) and Self-Organization Maps (SOM, parameters G 1-5, A 1-10, [15]) were constructed using GeneCluster software (M. Eisen). 3,000 transcripts were selected to be included in the analyses based on the differential expression from the mean. The deviation from the mean was calculated from the RMA values of the 45,000 probesets and the data were ranked in decreasing order to extract the first 3,000 genes. Only deviations equal or above 0.585 (1.5 fold change) and equal or below -0.585 (-1.5 fold change) were considered as significant.

Supervised learning: Significance Analysis of microarrays (SAM, [16]). SAM analyses were performed using Flexarray software using the normalized data of the 45,000 probesets. Data with p-values equal or below 0.01 and False Discover Rates (Benjamini Hochberg) equal or below 0.20 were included in further analyses. The data were ranked in decreasing order of the SAM d-score to obtain the list of the differentially expressed genes.

The NetAffx website (Affymetrix, Santa Clara, CA, USA) was used to retrieve gene ontology (GO) annotations, probe sequences, and was utilized as a link to Unigene (NCBI) for further functional studies.

The microarray dataset was deposited at Gene Expression Omnibus under accession number [GSE12581].

### Cell line and differentiation assay

The murine erythroleukaemic cell line HB22.2 was obtained from murine erythroblasts infected with Friend Murine Leukaemia Virus (F-MuLV) [17]. This cell line was maintained in alpha minimum essential medium (α-MEM) supplemented with 10% (FBS) (Invitrogen, Frederick, MD) plus a penicillin/streptomycin cocktail. To induce differentiation, HB22.2 cells were incubated in the presence of hemin (Sigma-aldrich H5533) at a concentration of 100 μM. The cells were harvested 24 hours and 72 hours after addition of hemin. K562 (ATCC, USA), HEL (ATCC, USA), Jurkat (ATCC, USA) and Tk6 (ATCC, USA) cells were grown in RMPI supplemented with 10% FBS plus a penicillin/streptomycin cocktail. MEG-01 (ATCC, USA), CMK (DSMZ, Germany) and LAMA84 (DSMZ, Germany) cells were grown in RPMI supplemented with 20% FBS plus a penicillin/streptomycin cocktail with a concentration of 10^5 ^cells/ml.

### RT-PCR

Oligo d(T) primed reverse transcription was performed using Omniscript Reverse Transcriptase (QIAGEN, Mississauga, Canada) in a 20 μl reaction volume (42°C, 1 h) by taking equal amounts of RNA (100 ng) from the Graffi-leukaemic cells and the murine HB22.2 cell line. cDNA (4 μl) was amplified using Taq polymerase (QIAGEN, Mississauga, Canada) at 94°C for 5 min, 72°C for 45 s, 57°C for 45 s, 72°C for 45 s, 72°C for 10 min. 25 and 28 cycles were used for the Graffi-leukaemic cells and 27 cycles were used for the HB22.2 cell line. 0.01 μl of cDNA and 25 cycles were used to amplify ubiquitously expressed *β-actin *and *Gapdh *genes. cDNA from the human haematopoietic cell lines was amplified using 500 ng of total RNA and the PCR reactions were performed using 4 μl of cDNA and 30 PCR cycles. Ubiquitously expressed human *GAPDH *gene was amplified using 0.01 μl of cDNA and 25 cycles. The primer sets are listed in supplementary data (Additional file 1). PCR products were analyzed on 2% agarose gels containing 0.5 μg/ml ethidium bromide. The gels were scanned (Molecular Dynamics Phosphorimager) and the density of the RT-PCR bands were assessed using the Quantity One software.

### Amplification of retroviral integration sites

This protocol was adapted from A. Berns and colleagues [18]. Tumor DNA from the spleen (10 μg) was digested with BamHI (New England Biolabs, Pickering, Canada). A splinkerette adaptor was generated by annealing 2 oligonucleotides (5'CATGGGCTAAAGAGGACTAATAACAAGCGTGGCTGAATGAGACTGGTGTCGA CACTAGTGG3', 5'GATCCCACTAGTGTCGACACCAGTCTCTAATTTTTTTTTTC AAAAAAA3', 95°C 5 min, cool-down to room temperature). 1 μg of digested DNA was ligated to the splinkerette oligonucleotide (molar ratio DNA/splinkerette 1:10) using a T4 DNA ligase (New England Biolabs, Pickering, Canada). The ligated product was then digested with ClaI (New England Biolabs, Pickering, Canada). A PCR followed by a nested PCR (150 ng of the ligated product) were performed using a primer located in the Graffi virus U3 sequence (5'GGTCTCTTGAAACTGCTGAGGG 3' and 5'GACCTTGATCTGAACTTCCCTATTC3') and one corresponding to the splinkerette oligonucleotide. The PCR program was the following: 94°C for 1 min, 68°C for 30 s (2 cycles), 94°C for 15 s, 58°C for 30 s, 72°C 3 min (27 cycles), 72°C 10 min. The PCR products were cloned (PCR Cloning Kit, Qiagen, Mississauga, Canada,) and sent for sequencing (Genome Quebec Innovation Centre, Montreal, Canada). The sequences were subjected to BLAST analysis against the annotated mouse genome database using Ensembl Genome Browser (release 45).

## Results

### Erythro- and megakaryoblastic leukaemias induced by the murine Graffi retrovirus and hybridization on microarrays

NFS newborn mice inoculated with the Graffi murine retrovirus develop an average of 20% of erythroleukemia and 20% of megakaryoblastic leukemias with a latency of about 148 days [12]. These mice suffer from severe anaemia and hepatosplenomegaly. The erythroleukaemic cells, Ter119^+^CD71^+^, and the megakaryoblastic leukaemic cells, CD41^+^Kit^+ ^or CD41^-^Kit^+^, are mainly found in the bone marrow and spleen of the diseased mice [12]. As opposed to Graffi-lymphoid leukaemias, the presence of blast cells is rare in blood smears of both erythro- and megakaryoblastic leukaemias, consistent with clinical data on human acute erythroleukaemia [8].

**Figure 1:**
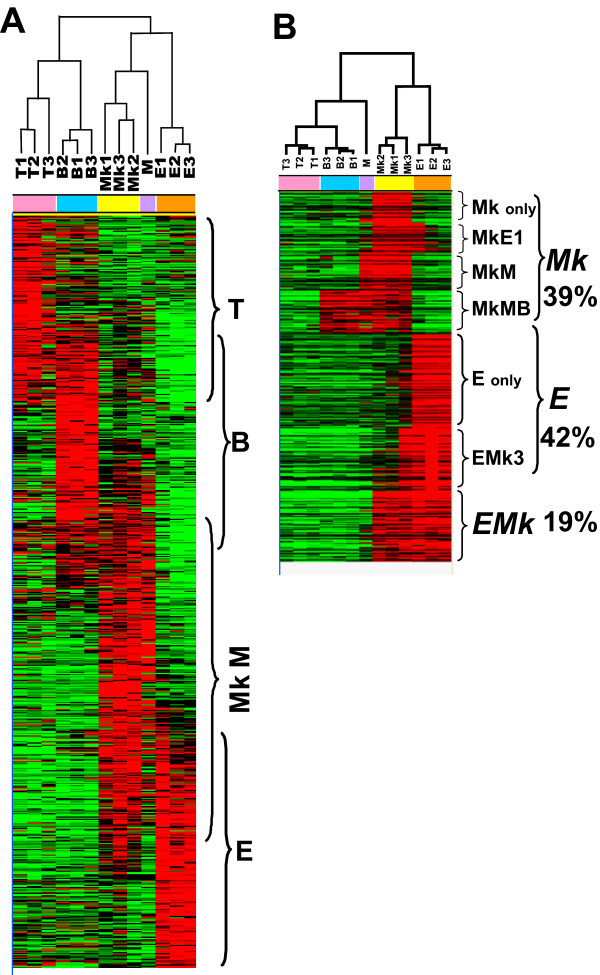
**Hierarchical clustering and data scatter**. (A) Heat map of the hierarchical clustering and SOM analyses of all data. (B) Heat map of the clustering and SOM analyses of the erythroid and megakaryoblastic over-expressed genes. Each column represents a leukaemia (T-cell: T1, T2, T3; B-cell: B1, B2, B3; Myeloid: M; Erythroid: E1, E2, E3; Megakaryoblastic: Mk1, Mk2, Mk3) and each row is assigned to a transcript. A red colour means a positive deviation of 0.595 and above from the mean (over-expression) and a green colour means a negative deviation of -0.595 and below from the mean (under-expression). The black colour corresponds to values comprised between 0.595 and -0.595. The result of the clustering on the arrays is shown as a dendrogram on the top of the figure. The nodes of the tree indicate the degrees of similarity between the leukaemias.

To gain insight into the cancerous signatures of the different leukaemias induced by Graffi MuLV, microarray experiments were designed to compare the expression signature of genes from each type of leukaemia. Cells from the infiltrated haematopoietic organs of the leukaemic mice were isolated (Additional file 2) and subjected to microarray analysis. Unsupervised learning methods, hierarchical clustering, and SOM analyses were used to uncover the primary pattern in the data (Figure 1). Altogether, four distinct gene clusters representing T-cells (T), B-cells (B), megakaryoblastic/myeloid cells (Mk/M) and erythroid cells (E) emerged from the clustering (Figure 1A).

A more detailed SOM analysis performed on the erythroid and megakaryoblastic genes further classified them into 3 major signatures: erythroid-megakaryoblastic EMk (19%), erythroid E (42%) and megakaryoblastic Mk (39%) (Figure 1B). E and Mk represent the genes over-expressed in the erythro- and megakaryoblastic leukaemias, respectively, and EMk represents the genes specifically over-expressed in both types. MkE1 indicates genes over-expressed in the 3 megakaryoblastic leukaemias (Mk1-Mk3) and the erythroleukaemia E1. Similarly, EMk3 corresponds to genes over-expressed in 3 erythroleukaemias (E1-E3) and the megakaryoblastic leukaemia Mk3. These results indicate that the leukaemias E1 and Mk3 are biphenotypic and express both erythroid and megakaryoblastic markers, which was previously observed in some Graffi-induced erythro- and megakaryoblastic leukaemias [12]. The MkMB signature includes genes over-expressed in megakaryoblastic leukaemias (Mk1-Mk3), myeloid leukaemia (M) and B-cell leukaemias (B1-B3). A detailed analysis of Mk, E and EMk signatures has revealed that many of the genes have not yet been reported in relation to the erythroid or the megakaryocytic lineages or to the corresponding leukaemias. The complete lists of genes detailing these non-lymphoid signatures are publicly available at http://www.biomed.uqam.ca/rassart/microarray.html[19].

The lineage specific expression of genes involved in heme biosynthesis, the megakaryocytic fibrinogen receptors and the expression of well known transcription factors validate the true lineage of these erythro- and megakaryoblastic leukaemias (Table 1).

**Table 1:** Genes specific to the erythroid and megakaryoblastic leukaemias

probeset	gene	SAM results *		Leukaemic samples**												
		d-score	FDR	T1	T2	T3	B1	B2	B3	M	E1	E2	E3	Mk1	MK2	Mk3
Fibrinogen receptor and related genes																
1417758_at	*Itga2b*	10.58	<0.001	-2.7	-1.6	-2.6	-2.9	-2.6	-2.7	-1.6	3.8	0.8	1.1	3.6	3.6	3.8
1421511_at	*Itgb3*	-	>0.01	-1.0	-0.7	-0.7	-0.8	-0.7	-0.9	-0.6	1.6	-0.7	-0.7	-0.6	2.9	3.1
1416066_at	*Cd9*	5.5	0.002	1.1	-2.5	2.5	-3.5	-3.6	-3.6	0.6	1.9	-1.8	-2.1	3.0	4.4	3.5
1456085_x_at	*Cd151*	7.56	<0.001	-1.2	-1.1	-0.7	-0.7	-0.9	-0.6	-0.3	1.4	-0.2	-0.3	1.8	0.8	2.1
1420558_at	*Selp*	12.03	<0.001	-1.0	-1.2	-1.9	-1.6	-1.5	-1.7	-1.1	-0.9	-1.7	-1.6	4.3	5.7	4.1
1457782_at	*Tln1*	7.19	<0.001	-0.7	-0.4	-1.4	-0.3	-0.8	-1.0	0.9	2.3	-1.5	-0.5	0.8	1.4	1.3
1424595_at	*F11r*	5.58	0.002	-0.9	-0.8	-0.9	-0.8	-0.6	-1.0	-0.6	0.2	-0.6	-0.6	2.0	3.1	1.5
1451097_at	*Vasp*	5.24	<0.001	-0.8	-1.3	0.1	0.3	-0.3	0.3	0.7	0.0	-0.6	-1.0	0.8	1.2	0.7
1418261_at	*Syk*	16.04	<0.001	-2.4	-2.8	-2.8	2.9	2.5	2.8	1.4	-1.5	-2.0	-3.1	1.5	1.6	1.8
1455349_at	*Rap1b*	4.63	<0.001	-0.7	-0.8	-1.1	-0.6	-1.2	-1.0	-0.2	2.1	-0.3	0.9	0.7	0.9	1.4
Heme biosynthesis																
1451675_a_at	*Alas2*	8.85	<0.001	-3.4	0.3	-0.9	-2.5	-3.8	-3.2	-1.3	4.9	4.9	4.8	-2.8	-0.5	3.5
1424877_a_at	*Alad*	8.91	<0.001	-1.2	-0.4	0.0	-1.3	-1.6	-0.7	-1.6	2.2	2.2	2.0	0.4	-0.9	1.2
1426475_at	*Hmbs*	13.44	<0.001	-1.2	-1.0	-0.4	-1.5	-1.1	-1.1	-1.1	2.6	3.4	2.7	-0.8	-0.9	0.5
1423482_at	*Uros*	-	-	-0.6	-0.3	-0.2	-0.3	0.1	0.2	-0.9	0.7	1.5	0.2	0.0	-0.5	0.0
1417206_at	*Urod*	10.87	<0.001	0.0	-0.4	-0.3	-1.2	-0.9	-1.0	-0.3	1.8	1.6	1.4	-0.6	-0.5	0.3
1422493_at	*Cpox*	22.18	<0.001	-1.6	-1.2	-0.2	-0.5	-0.8	-1.0	-1.5	2.7	3.1	3.2	-1.0	-0.7	-0.4
1416618_at	*Ppox*	4.58	0.004	-0.5	-0.1	-0.3	0.1	-0.2	-0.4	0.0	0.7	1.4	0.6	-0.6	-0.4	-0.4
1418699_s_at	*Fech*	8.61	<0.001	-0.7	-1.0	-0.3	-1.9	-1.9	-2.0	-0.4	2.5	3.1	2.2	-0.7	0.0	1.1
Erythroid and megakaryoblastic transcription factors																
EMk																
1450333_a_at	*Gata2*	7.4	0.01	-1.9	-1.6	-1.9	-1.7	-1.8	-1.9	-1.1	-0.6	2.2	0.6	3.4	3.4	2.8
1423603_at	*Zfpm1(Fog1)*	5.4	0.02	-1.2	-1.7	-1.4	0.1	-0.6	-0.2	-3.9	2.0	2.0	2.0	1.3	0.8	0.9
1449389_at	*Scl/Tal1*	20.8	<0.01	-3.3	-2.5	-3.1	-3.2	-2.5	-2.5	-2.5	4.1	3.8	3.5	2.6	2.9	2.9
1454086_a_at	*Lmo2*	4.5	0.03	-5.1	-3.8	-4.6	0.7	-1.3	-0.9	0.7	2.8	3.0	2.9	1.3	2.5	1.8
1452001_at	*Nfe2*	8.3	0.01	-4.2	-3.0	-3.4	-3.8	-3.0	-3.2	1.3	3.7	3.5	3.3	2.5	3.7	2.8
EMk3																
1449232_at	*Gata1*	4.6	0.03	-1.9	-1.9	-2.1	-2.1	-2.3	-2.7	-1.9	4.5	4.1	4.5	0.6	-1.4	2.5
MkE1																
1441584_at	*Fli1*	5.3	0.1	-0.2	-0.4	-0.5	0.7	0.6	0.5	0.7	2.6	-4.8	-4.9	1.8	2.4	1.6
Erythroid transcription factors																
EMk3																
1418600_at	*Klf1*	6.8	0.04	-2.9	-2.3	-2.4	-2.7	-2.7	-2.8	-1.8	4.8	5.4	5.3	1.7	-1.7	2.1
1419311_at	*Trim10*	8.9	0.02	-2.0	-1.4	-2.1	-2.1	-2.1	-2.0	-1.5	5.0	4.1	3.8	-1.0	-0.8	2.1
Megakaryoblastic transcription factors																
MkE1																
1421461_at	*Mpl*	16.1	<0.001	0.07	-1.9	-1.6	-2.1	-2.0	-2.1	-2.0	-1.3	1.3	-1.0	-1.7	4.9	4.4
MkE1																
1440878_at	*Runx1*	4.5	0.11	0.6	-1.8	-1.0	0.2	0.2	0.5	-0.5	0.9	-0.8	-0.1	0.7	0.8	0.3

* SAM p-value less than 0,001 for every sample

** amplitude of deviation from the mean calculated from the RMA values

### The megakaryoblastic signature

The megakaryoblastic specific genes assigned with a functional annotation (GO terms) were divided into different functional classes. Table 2 lists some genes potentially implicated in the disease but the complete data are readily available [19]. Within this list of genes, the oncogenes *Meis1 *(*Myeloid ecotropic viral integration site 1*), *Pbx1 *(*Pre B-cell leukaemia transcription factor 1*), *Evi1 *(*ecotropic viral integration site 1*), *Evi3 *(*Zfp521*, *zinc finger protein 521*) and the co-repressor *Cbfa2t3h *(*Core-binding factor, runt domain, alpha subunit 2, translocated to, 3 (human), Eto2*) have already been related to megakaryoblastic leukaemias or megakaryocytic lineage [20-25].

**Table 2:** Genes over-expressed in the megakaryoblastic leukaemias

Probeset	Gene	SAM results			Leukaemic samples *													GSE 6593
		d-score	p-value	FDR	T1	T2	T3	B1	B2	B3	M	E1	E2	E3	Mk1	Mk2	Mk3	
Genes potentially implicated in the disease **																		
Mk																		
1443260_at	*Meis1*	12.0	<0.001	0.1	-1.5	-1.3	-1.7	-1.5	-1.3	-1.7	-1.4	-1.4	-1.3	-1.3	4.5	5.9	4.0	ND
1428647_at	*Pbx1*	10.2	<0.001	0.07	-1.6	-1.1	-1.1	-1.3	-1.3	-1.6	-1.2	-1.2	-1.1	-1.4	4.3	5.3	3.3	ND
1417155_at	*Nmyc*	16.0	<0.001	0.08	-2.0	-1.6	-1.9	-0.7	-0.6	-1.2	-1.6	-1.8	-1.5	-1.9	4.8	5.6	4.4	NS
1451332_at	*Evi3*	13.0	<0.001	0.08	-1.1	-1.3	-1.1	-1.1	-1.0	-0.9	-0.7	-0.7	-0.6	-0.9	3.5	3.3	2.6	NS
1441350_at	*Fgf3*	5.4	0.002	0.12	-0.7	-0.7	-0.6	-0.6	-0.7	-0.8	-0.6	-0.4	-0.6	-0.5	1.3	2.9	1.9	↓
1417552_at	*Fap*	7.2	<0.001	0.09	-1.7	-1.6	-1.7	-1.8	-1.7	-1.7	-1.7	-1.5	-1.6	-1.4	5.9	7.0	3.6	ND
1438325_at	*Evi1*	4.5	0.005	0.16	-1.8	-0.9	-1.4	-1.7	-1.7	-1.4	-1.8	-1.1	-1.4	-1.5	7.3	2.4	4.8	ND
1440964_s_at	*Cbfa2t3h*	11.0	<0.001	0.08	-0.4	-0.7	-1.1	-0.3	-0.6	-0.3	-0.1	0.2	-0.6	-0.2	1.4	1.4	1.2	NS
MkE1																		
1452072_at	*Myct1*	7.2	<0.001	0.07	-2.0	-1.7	-1.7	-1.6	-1.5	-1.6	-1.4	2.0	-1.1	0.6	3.0	4.5	2.5	NS
MkMB																		
1437247_at	*FosL2*	10.3	<0.001	0.01	-0.8	-0.5	-0.8	-0.7	-0.7	-0.8	1.8	-0.5	-0.5	-0.6	1.6	1.6	0.8	ND
1417409_at	*Jun*	5.1	<0.001	0.03	-0.3	-1.4	-3.3	1	1.1	1.4	4.1	-1.3	-1.5	-2.3	0.8	0.8	0.91	↓
1434705_at	*Ctbp2*	15.3	<0.001	0.01	-1.1	-1.0	-1.6	-0.9	-1.1	-1.3	3.2	-1.2	-1.4	-1.9	3.3	2.7	2.4	NS
1452514_a_at	*Kit*	10.5	<0.001	0.01	-2.2	-2.1	-2.2	-2.2	-2.3	1.2	2.8	-1.7	-1.6	-2.2	3.8	4.6	4.0	↓
1418747_at	*Sfpi1*	9.9	<0.001	<0.01	-2.6	-1.7	-1.7	1.4	0.9	1.1	3.0	-1.7	-1.4	-1.8	1.2	2.1	1.1	↓
1452410_a_at	*Fes*	10.2	<0.001	<0.01	-2.3	-1.7	-1.7	1.8	0.5	1.0	3.0	-1.4	-2.0	-1.9	1.4	2.2	1.1	↓
1428669_at	*Bmyc*	5.8	<0.001	0.04	0.8	-0.9	0.9	-1.5	-1.2	-0.5	1.3	-1.6	-1.2	-1.4	1.7	2.2	1.4	↓
1420710_at	*Rel*	5.8	<0.001	0.02	-0.4	0.0	-0.8	0.4	1.0	0.9	1.0	-0.9	-1.3	-1.1	0.6	-0.1	0.6	NS
Genes potentially implicated in inflammation response																		
MkMB																		
1449222_at	*Ebi3*	11.2	<0.001	0.01	-1.4	-1.3	-1.2	-1.4	-1.2	-1.3	3.6	-0.7	-1.1	-1.6	2.2	3.1	2.23	↓
1418262_at	*Syk*	1.6	<0.001	<0.01	-2.4	-2.5	-2.8	2.8	2.4	2.6	1.5	-1.6	-2.2	-2.8	1.4	1.6	1.96	NS
1419132_at	*Tlr2*	6.7	<0.001	0.01	-0.8	-1.4	-1.6	0.6	0.1	0.6	2.4	-0.9	-1.4	-1.5	1	2	0.9	↓
1418163_at	*Tlr4*	6.3	<0.001	0.01	-1.8	-1.6	-1.4	0.5	1.8	2.2	2.5	-1.2	-0.8	-1.3	0.4	0.7	0.0	ND
1456046_at	*C1qr1*	8.1	<0.001	<0.01	-2.2	-3.7	-4.2	4.1	3.9	4.1	3.7	-2.3	-2.2	-4.1	0.9	0.6	1.4	ND
Genes selected for RT-PCR validation																		
1434141_at	*Gucy1a3*	18.2	<0.001	0.07	-1.2	-0.6	-1.0	-1.0	-1.1	-1.2	-1.0	0.2	-1.1	-1.0	3.0	3.2	2.7	-
1437687_x_at	*Fkbp9*	23.7	<0.001	0.07	-1.4	-1.3	-1.6	-1.3	-1.3	-1.3	-1.1	-1.0	-1.0	-1.1	4.1	4.6	3.9	NS
1453771_at	*Gulp1*	14.2	<0.001	0.07	-0.7	-0.7	-0.5	-0.6	-0.9	-0.9	-0.9	-1.0	-0.9	-0.9	3.1	2.6	2.3	-
1448561_at	*Ncf2*	18.5	<0.001	<0.01	-2.6	-2.4	-2.1	2.0	1.6	1.6	3.0	-2.1	-2.2	-2.4	1.6	2.5	1.6	NS
1450333_a_at	*Gata2*	7.4	<0.001	0.01	-2.3	-2.0	-2.3	-2.1	-2.4	-1.9	-1.6	0.2	2.4	1.1	3.6	4.0	3.4	↓

* amplitude of deviation from the mean calculated from the RMA values

** cell cycle/cell growth/development/angiogenesis/DNA repair/transcription regulation

ND (not determined), NS (not significant)

However, several other genes, for example *Nmyc *(*Neuroblastoma myc-related oncogene 1*), *Fgf3 *(*Fibroblast growth factor 3*) and *Fap *(*Fibroblast activation protein*), that are also known oncogenes [26-29], have never been reported with megakaryoblastic leukaemias. *Myct1 *(*Myc target 1*) is also potentially implicated in the disease as it is positively regulated by *Myc *and contains tumorigenic properties by itself [30].

Among the list of genes identified as the MkMB signature, we found *Jun *(*Jun oncogene*), *Fosl2 *(*Fos-like antigen 2*) and *Fes *(*Feline sarcoma oncogene*) (Table 2, [31,32]). The co-repressor *Ctbp2 *(*C-terminal binding protein 2*), *Sfpi1 *(*SFFV proviral integration 1*, *PU.1*) and *Kit *(*Kit oncogene*) are also MkMB signature elements. *Ctbp2 *is known to interact with Evi1 and Fog [33]. *Sfpi1 *was shown to regulate the expression of the *integrin αIIb *(*Itga2B*, CD41) in a TPO-induced Mpl-UT7 model [34,35] and it was reported as an insertional target of the Graffi MuLV [36].

Due to technical limitations, no megakaryoblastic control was present in our study. Normal megakaryocytes and therefore megakaryoblasts represent a minor population in normal mice and it was not possible to obtain enough purified cells with the technique utilized for other samples. We therefore compared our dataset to a study of murine megakaryocytic maturation indicating up- or down-regulation during differentiation (dataset GSE6593, [37]). According to the megakaryocytic GSE6593 dataset, *Fgf3 Jun*, *Kit*, *Sfpi, Fes *and *Bmyc *are down-regulated upon megakaryocytic differentiation (Table 2).

When genes within the MkMB signature were compared to GO annotations, one gene class was over-represented. Many of these genes were membrane receptors and extra-cellular factors known to be expressed by antigen presenting cells (APC) as well as implicated in inflammatory response [19]. For example, *Tlr2 *(*Toll-like receptor 2*), *Tlr4 *(*toll-like receptor 4*), *Syk *(Spleen tyrosine kinase) and *Ebi3 *(*Epstein-Barr virus induced gene 3*) are part of the Toll-like receptor signaling pathway to respond to microbial stimuli (LPS) and induce inflammation (Table 2). Confirming our data, *Tlr2*, *Tlr4 *and *Syk *are already known to be expressed by the megakaryocytic lineage [38,39].

**Figure 2:**
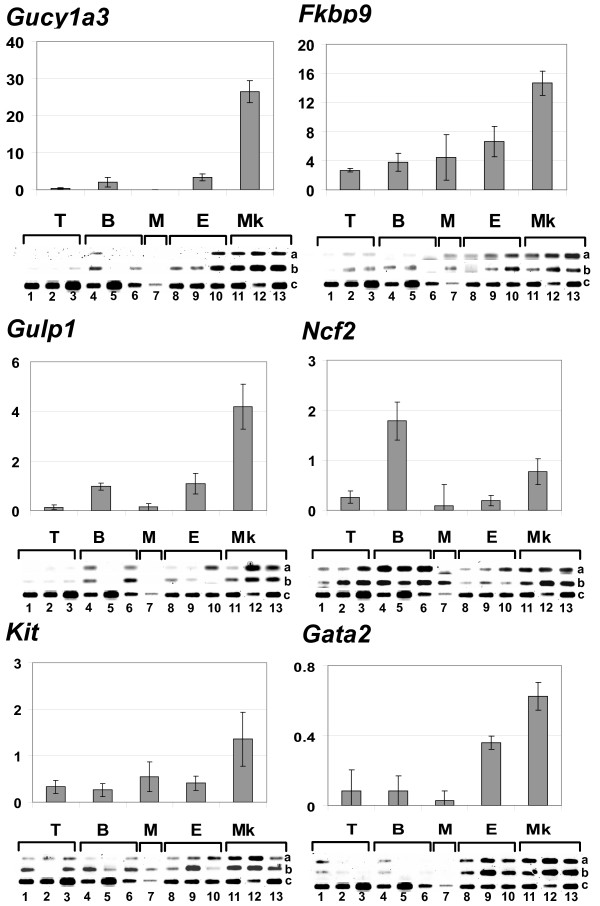
**Megakaryoblastic specific genes**. Semi-quantitative RT-PCR analysis in 3 T-cell (T, 1-3), 3 B-cell (B, 4-7), 1 myeloid (M, 8), 3 erythroid (E, 8-10) and 3 megakaryoblastic leukaemias (Mk, 11-13). RT-PCRs have been performed in quadruplicate for each sample. Two out of 4 RT-PCR runs are shown for the leukaemic samples (lane a: 25 PCR cycles; lane b: 28 PCR cycles) and 1 out of 4 are shown for the β-actin internal control (lane c). The histograms represent the quantification of the density of the bands relative to the β-actin sample including all RT-PCR runs for each type of leukaemia (T, B, M, E, Mk).

To validate the microarray data, the expression of several megakaryoblastic specific genes was tested by semi-quantitative RT-PCR in samples different from those analyzed in the microarray experiments (Table 2 and Figure 2). Within these genes, *Kit *and *Gata2 *were tested due to their important roles in haematopoiesis. The other genes were selected for experimentation since they had no prior association with megakaryocytic lineages or with the corresponding leukaemia and also since their function remained poorly studied (Table 2, 'Genes selected for RT-PCR validation'). Within these genes, *Gulp1 *(*Engulfment adaptor PTB domain containing 1*) and *Gucy1a3 *(*Guanylate cyclase 1, soluble, alpha 3*) gave the highest specificity in the RT-PCR experiments with a strong expression in the 3 megakaryoblastic leukaemias (Figure 2). Most of the non-megakaryoblastic leukaemias showed very low or no expression of these genes. *Ncf2 *(*Neutrophil cytosilic factor 2*) is highly expressed in the B-cell and megakaryoblastic leukaemias in accordance with the microarray data (Table 2). *Fkbp9 *(*FK506 binding protein 9*) is strongly expressed in the megakaryoblastic leukaemias with a weaker but sustained expression in other types of leukaemias. *Gata2 *is strongly expressed in the megakaryoblastic leukaemias and to a lower level in the 3 erythroleukaemias. Finally, *Kit *was amplified in all leukaemias but with the strongest expression in the megakaryoblastic ones (Figure 2).

### The erythroid signature

The results of the erythroid signature were compared to the transcriptome analysis of G1E cells during *GATA1 *induced differentiation (dataset GSE628, [40]). Our results correlate neatly with this dataset although the Affymetrix Genechip MG-U74A used by Welch and colleagues contains only one third the probes of the MOE 430.2 used in our study. In the Welch *et al *study, the genes that displayed increased expression during differentiation tended to be under-expressed in the Graffi-induced erythroleukaemias compared to the control sample and vice-versa. This suggests that the Graffi-induced erythroleukaemias are blocked in an earlier stage than the control sample taken from a population of Ter119^+^CD71^+ ^erythroblasts in the bone marrow. Table 3 provides examples of this correlation between the two studies. For example, *Car1 *(*Carbonic anhydrase 1*) is over-expressed in the leukaemias in comparison to the control (positive value, column 'E-CE') and its expression decreases during erythroid differentiation (descending arrow, column 'G1E'). *Alas2 *(*Aminolevulinic acid synthase 1*), involved in the heme biosynthesis, is under-expressed in the leukaemias in comparison to the control and its expression increases during erythroid differentiation.

**Table 3:** Genes specific to the erythroid leukaemias

Probeset	Gene	SAM results			Leukaemic samples*														
		d-score	p-value	FDR	T1	T2	T3	B1	B2	B3	M	E1	E2	E3	Mk1	MK2	Mk3	E-CE	G1E
Correlation with the G1E database																			
1416193_at	*Car1*	8.2	<0.001	0.02	-3.4	-2.1	-3.2	-3.0	-3.0	-3.2	-2.4	6.0	5.9	1.7	1.4	-2.1	3.0	1.2	↓
1422316_at	*Gp1ba*	12.7	<0.001	0.01	-2.5	-2.1	-1.9	-1.9	-2.0	-2.4	-1.9	4.3	3.3	1.7	0.1	-0.1	3.7	2.9	↓
1422817_at	*Gp5*	7.5	<0.001	0.03	-3.6	-2.3	-3.0	-3.0	-2.9	-2.8	-1.9	4.5	3.6	1.7	2.4	1.6	3.4	3.8	↓
1424968_at	*2210023G05Rik*	12.3	<0.001	0.01	-2.4	-2.3	-2.1	-2.2	-2.2	-1.9	-1.9	4.4	5.5	5.1	-0.2	-1.1	1.4	1.3	↓
1425677_a_at	*Ank1*	18.2	0.001	0.01	-1.5	-1.3	-1.3	-1.5	-1.1	-0.9	-1.0	3.1	3.6	3.3	-0.5	-1.1	0.1	-0.8	↑
1416464_at	*Slc4a1*	11.6	<0.001	0.02	-2.3	-1.4	-2.0	-2.4	-2.7	-2.0	-1.2	4.7	4.6	1.7	-1.8	0.2	3.2	-2.4	↑
1451675_a_at	*Alas2*	11.0	<0.001	0.02	-3.4	0.3	-0.9	-2.5	-3.8	-3.2	-1.3	4.9	4.9	1.7	-2.8	-0.5	3.5	-1.1	↑
1418699_s_at	*Fech*	8.9	<0.001	0.02	-0.8	-1.0	-0.7	-1.4	-1.7	-1.5	-0.5	2.2	2.5	1.7	-0.2	0.0	1.3	-2.4	↑
Genes potentially implicated in the disease ***																			
E																			
1417514_at	*Ssx2ip*	13.97	<0.001	0.01	-1.1	-0.8	-1.4	-0.3	-0.3	-0.5	-0.5	2.8	2.7	3.0	-2.4	-1.4	0.3	1.1	↓
1460057_at	*Gdf3*	9.4	<0.001	0.01	-1.3	-1.2	-1.0	-1.3	-0.9	-1.0	2.0	2.2	2.4	2.8	-1.3	-1.3	-0.1	2.9	NS
1419665_a_at	*Nupr1*	8.7	<0.001	0.01	-1.5	-1.5	-1.3	-1.3	-1.3	-1.7	0.3	3.9	2.5	3.3	-0.9	-0.6	0.2	4.0	-
1443969_at	*Irs2*	6.5	<0.001	0.02	0.1	-0.1	-1.5	-0.2	-0.4	0.4	-1.0	1.1	2.1	1.4	-1.2	-0.5	-0.2	1.2	↑
1417165_at	*Mbd2*	17.38	<0.001	0.01	-0.7	-0.1	-0.4	0.0	-0.1	-0.3	-0.6	1.3	1.3	1.3	-0.6	-0.7	-0.2	0.9	-
1449256_a_at	*Rab11a*	10.59	<0.001	0.01	-1.0	-0.3	-1.2	-0.6	-0.6	-0.3	-0.4	1.7	1.7	1.3	-0.3	-0.1	0.2	1.7	-
1417396_at	*Podxl*	6.5	<0.001	0.02	-0.8	-0.9	-1.0	-1.0	-0.9	-0.7	-1.0	1.3	1.6	1.7	1.6	0.2	-0.1	2.4	NS
EMk3																			
1422737_at	*Ncoa3*	4.1	0.003	0.10	-0.3	-1.2	-0.1	-0.5	-0.4	0.3	-0.3	1.5	0.4	0.8	0.0	-0.7	0.6	2.2	NS
1435458_at	*Pim1*	5.4	<0.001	0.06	-1.0	-1.3	-2.0	-0.6	-0.4	-0.4	-0.2	1.5	1.5	1.0	0.1	0.9	0.9	0.6	↑
Genes selected for RT-PCR validation																			
1449232_at	*Gata1*	4.58	<0.001	0.03	-1.9	-1.9	-2.1	-2.1	-2.3	-2.7	-1.9	4.5	4.1	4.5	0.6	-1.4	2.5	0.4	↑
1425571_at	*Slamf1*	9.9	<0.001	0.02	-1.3	-1.6	-1.7	-1.2	-1.6	-1.5	-2.0	4.1	3.0	1.7	-0.3	-1.2	1.9	4.8	-
1418493_a_at	*Snca*	10.2	<0.001	0.02	-3.3	-0.2	-1.0	-2.5	-3.2	-1.8	-1.5	4.6	4.2	1.7	-1.0	-0.7	2.7	-2.6	↑
1418061_at	*Ltbp2*	15	<0.001	0.01	-0.7	-0.7	-0.7	-0.7	-0.8	-0.7	-0.9	2.2	1.5	1.7	-0.5	-0.8	1.4	2.0	NS
1419069_at	*Rabgef1*	25.0	<0.001	0.01	-0.5	-0.8	-0.4	-0.9	-1.3	-1.2	-0.8	2.2	2.3	2.3	-0.4	-0.4	-0.1	4.8	-
1427357_at	*Cda*	25.0	<0.001	0.01	-1.5	-1.6	-1.6	-1.7	-1.8	-0.6	-1.3	4.3	4.8	4.9	-1.6	-1.7	-0.7	6.0	-
1417152_at	*Btbd14a*	9.5	<0.001	0.02	-0.6	-0.9	-0.9	-0.9	-0.8	-0.9	-0.5	2.3	1.5	1.7	-0.4	-0.4	1.0	2.3	NS

* amplitude of deviation from the mean calculated from the RMA values

** ratio E-CE: mean of the deviation of E1, E2 and E3/erythroid control value

*** cell cycle/cell growth/development/angiogenesis/DNA repair/transcriptionregulation

Table 3 presents a summary of the erythroid specific genes over-expressed in comparison to the control sample and potentially implicated in the disease but the complete data are readily available [19]. Within these genes, *Gdf3 *(*Growth differentiation factor 3*), *Podxl *(*Podocalyxin-like*), *Nupr1 *(*Nuclear protein 1*), *Pim1 *(*Proviral integration site 1*) and *Isr2 *(*Insulin receptor substrate 2*) are known to be regulated by erythropoietin [41-43]. The oncogene *Pim1 *was found rearranged in Friend helper MuLV-induced erythroleukaemias and Graffi-induced leukaemias [36,44]. *Ssx2ip *(*Synovial sarcoma, X breakpoint 2 interacting protein*) was found over-expressed in some AML patients and is expressed by K562 erythroid cells [45]. *Rab11a *(*RAB11a, member RAS oncogene family*) was reported to regulate the recycling of the transferrin receptor [46]. This protein interacts with Evi5 [47] and has a potential role in cancer [48]. The oncogene *Ncoa3 *(*Nuclear receptor coactivator 3*) is over-expressed in numerous cancer types such as breast, prostate, ovarian, gastric, pancreatic and colorectal cancers [49].

**Figure 3:**
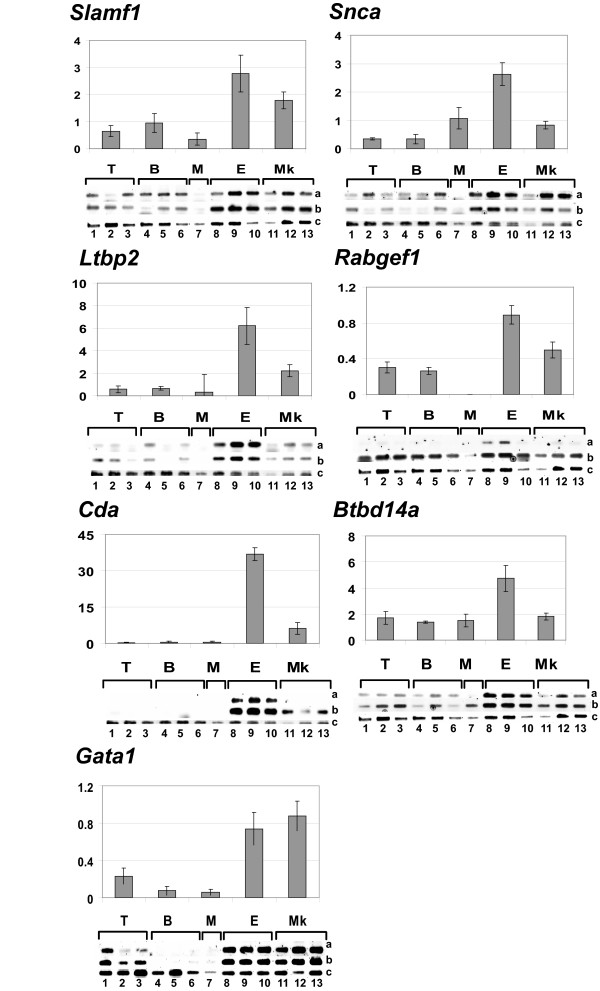
**Erythroid specific genes**. Semi-quantitative RT-PCR analysis in 3 T-cell (T, 1-3), 3 B-cell (B, 4-7), 1 myeloid (M, 8), 3 erythroid (E, 8-10) and 3 megakaryoblastic leukaemias (Mk, 11-13) was utilized for each of the 6 tested genes. RT-PCRs have been performed in quadruplicate for each sample. Two out of 4 RT-PCR runs are shown for the leukaemic samples (lane a: 25 PCR cycles; lane b: 28 PCR cycles) and 1 out of 4 are shown for the β-actin control sample (lane c). The histograms represent the quantification of the density of the bands relative to the β-actin sample including all RT-PCR runs for each type of leukaemia (T, B, M, E, Mk).

Many genes have not yet been reported in relation to erythroid leukaemias and several others have a still unknown function and some of them have been selected for RT-PCR validation (Table 3 and Figure 3). *Gata1 *was tested due to its important role in haematopoiesis (Figure 3). Among the 7 tested erythroid genes (Table 3, 'Genes selected for RT-PCR validation'), both *Cda *(*Cytidine deaminase*) and *Ltbp2 *(*Latent transforming growth factor beta binding protein 2*) showed a very high and specific expression in the 3 erythroleukaemias (Figure 3). *Slamf1 *(*Signaling lymphocytic activation molecule family member 1*), *Snca *(*Synuclein alpha*) and *Btbd14a *(*BTB/POZ domain containing 14A*) are higher expressed in the erythroleukaemias and lower in the megakaryoblastic leukaemias but were also amplified in other types of leukaemias. *Rabgef1 *is specifically expressed in the erythroleukaemias at 25 PCR cycles (lane a) but was amplified in all samples at 28 PCR cycles (lane b). *Gata1 *is equally highly expressed in the erythroid and in the megakaryoblastic leukaemias (Figure 3).

### RT-PCR validation in a Friend virus murine erythroleukaemia cell line

**Figure 4:**
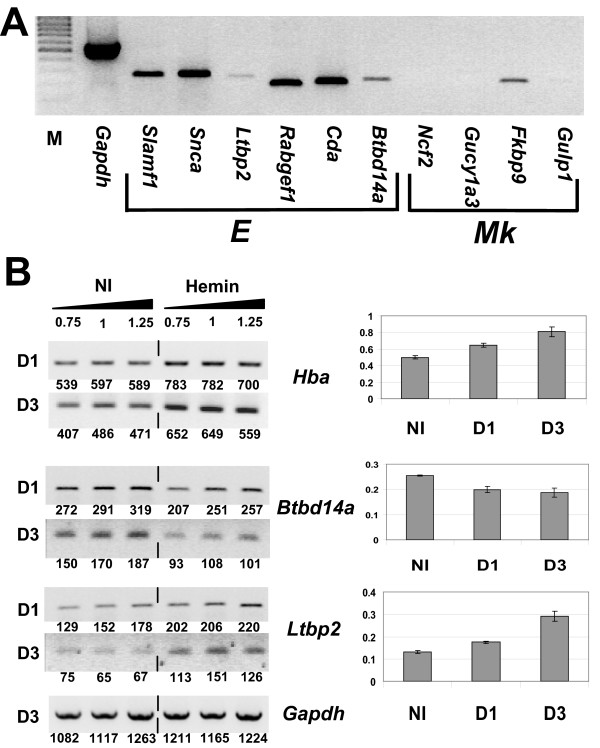
**Expression of the erythroid and megakaryoblastic specific genes in the murine erythroleukaemia cell line HB22.2 during hemin-induced differentiation**. (A) Semi-quantitative RT-PCR analysis of 6 erythroid specific genes (E) and 4 megakaryoblastic specific genes (Mk). The first lane (M) indicates the low-range DNA marker. (B) Hemin-induced differentiation assay of HB22.2 cells. Semi-quantitative RT-PCR analysis of Hba as differentiation control, Btbd14a and Ltbp2 was performed in non-induced (NI) and induced (Hemin) cells at day 1 (D1) and day 3 (D3) following hemin addition. *GAPDH *expression was used as internal control. Three independent RT-PCR reactions were performed on each sample using increasing amount of the RT reaction (0.75 volume, 1 volume, 1.25 volume). The band intensity was measured and indicated below each band. On the right half of the figure, the histograms show the average results of the band intensities relative to the Gapdh control sample.

The expression of the erythroid and megakaryoblastic specific transcripts validated by RT-PCR (Figures 2 and 3) was further assessed on a different erythroid model (Figure 4). The erythroleukaemia cell line HB22.2 has been derived from a leukaemia induced by the Friend Murine Leukaemia virus (F-MuLV) and it presents a very immature erythroid phenotype (Kit^+^CD71^+^Ter119^-^CD41^-^) ([17]). The 6 erythroid genes (*Slamf1*, *Snca*, *Ltbp2*, *Rabgef1*, *Cda *and *Btbd14a*) are expressed in HB22.2 but the intensity of the *Ltbp2 *and *Btdb14a *bands were weaker (Figure 4A). In accordance with our expectations, the megakaryoblastic genes, *Ncf2*, *Gucy1a3 *and *Gulp1*, could not be amplified. *Fkbp9 *is the only megakaryoblastic gene that gave a weak signal in this erythroid cell line. Indeed, *Fkbp9 *showed the strongest erythroid amplification in the RT-PCR validation experiment (Figure 2). Thus, these results show that, despite the close relationship between erythroid and megakaryoblastic leukaemias, this experiment's design enabled us to find genes that can distinguish these 2 types of murine leukaemias from each other.

We then induced HB22.2 differentiation with hemin and tested the expression of the erythroid specific genes at different time-points (Figure 4). Integration of F-MuLV upstream of Fli-1 is shown to block differentiation of this cell line [17]. However, these cells are able to undergo differentiation with hemin associated induction of alpha globin (Figure 4B). Among the 6 erythroid genes tested, both *Btbd14a *and *Ltbp2 *showed reproducible changes with a decrease and an increase with differentiation, respectively (Figure 4B). The increased expression of *Ltbp2 *indicates that it likely plays a role in mature erythroid cells whereas *Btbd14a *is likely to play a role in immature erythroid cells and this correlates well with a putative oncogenic role for this gene.

### Validation in human leukaemic cell lines

**Figure 5:**
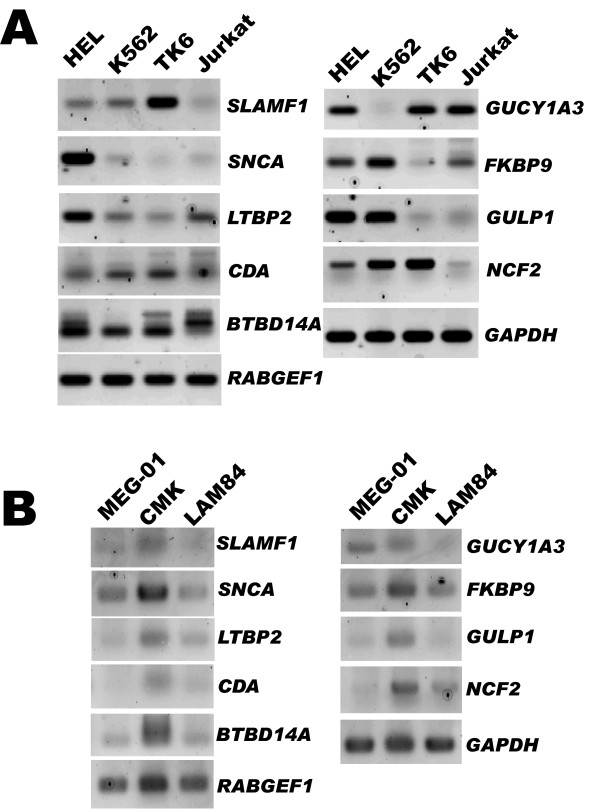
**Validation in human cell lines**. (A) RT-PCR analysis in the erythroid-like human cell lines K562 and HEL, the B- cell lymphoblastic cell line TK6 and the T-cell leukaemia cell line Jurkat. (B) RT-PCR analysis in the human megakaryoblastic cell lines MEG-01 and CMK and the human erythroid cell line LAMA-84. The left and right parts of each panel correspond to genes identified in the erythro- and megakaryoblastic leukaemias, respectively. *GAPDH *was used as internal control.

The proteins encoded by these erythroid and megakaryoblastic specific genes have high homologies with their human counterparts. This makes it likely that these human and murine proteins have the same functional role. Gene expression was first tested in 2 human erythroid-like cell lines, HEL and K562, a human B-cell leukaemia cell line, TK6, and a human T-cell leukaemia cell line, Jurkat (Figure 5A). Because HEL and K562 are known to harbour mixed myeloid lineage phenotype, the genes were further tested in 2 human megakaryoblastic cell lines, MEG-01 and CMK, and 1 erythroid cell line LAMA-84 (Figure 5B).

The results show that all tested erythroid and megakaryoblastic specific genes were amplified in some or all of the cell lines confirming their expression in human erythroid or megakaryoblastic cell lines. Moreover, several were also amplified in the lymphoid lines TK6 and/or in Jurkat. *RABGEF1 *and *BTBD14A *were the most ubiquitous genes with a strong amplification in lymphoid and non-lymphoid cell lines. *GULP1 *and *SNCA *were the most specific to the non-lymphoid lineage with no expression in TK6 or Jurkat cell lines.

### Viral integrations in the megakaryoblastic leukaemias

We also identified retroviral integration sites (RIS) in the 3 megakaryoblastic leukaemias (Mk1-3) in order to search for genes that may have contributed to the oncogenic transformation. Several RIS were amplified, cloned and sequenced in these 3 tumors (11 in Mk1, 5 in Mk2 and 10 in Mk3) (Table 4). No common integration sites (CIS) that could clearly indicate the contribution to the oncogenic events were found. Therefore, the results were compared to the retroviral tagged cancer gene database (RTCGD, [50]) that compiles the RIS identified in different murine cancer models (underlined in Table 4). Genes identified in multiple screens have a high probability of involvement in oncogenic transformation. Eleven genes near the RIS were found in the RTCGD (underlined in Table 4). Some of these genes, such as *Ccnd1 *and *Myc*, are largely known to be involved in leukaemia. *Foxf1 *is a transcription factor known to regulate the megakaryocytic integrin β3 (CD61) [51]. Interestingly, *Kit *and *Gata2 *are also part of RIS. The presence of the RIS near *Kit *in Mk1 and near *Gata2 *in Mk3 has been validated by PCR in the sorted leukaemic megakaryoblastic population (not shown). Two other genes, *Irf8 *and *Itga1 *targeted by a RIS (Table 4) are also of interest: *Irf8 *is not included in the RTCGD database but is a known CIS [52] and the *ITGA1 *locus is repressed by methylation during megakaryopoiesis in humans [53].

**Table 4:** Graffi-virus integration sites in the megakaryoblastic leukaemias

Sample	Chromosome	Genome	Upstream genes*				Integra-tion within a gene	Downstream genes*			
			**gene 1**	**kb**	**gene 2**	**kb**		**gene 1**	**kb**	**gene 2**	**kb**
			
**Mk1**	**1D**	**93018002**	*Gm817*	**17**	*Lrrfip1*	**60**	Ramp1**	*Ube2f*	**95**	*Scly*	**130**
	**1G2**	**153451217**	*Rnf2*	**188**	*1190005F20Rik*	**230**	*Niban*	*Edem3*	**92**	*Q8K3Do*	**199**
	**4B1**	**41816941**	*2310028H24Rik*	**133**	*1700066J25Rik*	**138**	*Dnaic1*	*Cntfr*	**29**	*Dcnt3*	**86**
	**5E 1**	**75741720**	*Pdgfra*	**216**	*Gsh2*	**382**	-	Kit**	**114**	*Kdr*	**472**
	**6F3**	**128801887**	*Klrb1b*	**10**	*Clec2h*	**159**	-	*Clec21*	**50**	*Klrb1f*	**208**
	**6G1**	**135264443**	*Gsg1*	**61**	*Hebp1)*	**130**	-	*Pbp2*	**10**	*Dynlt1*	**50**
	**11B4**	**70471623**	Pfn1**	**0.8**	*Rnf167*	**4**	-	*Eno3*	**1.7**	*Spag7*	**8.2**
	**11B5**	**80139013**	*Rhot1*	**55**	*Rnf135*	**123**	*Rhbd13*	*Zfp207*	**60**	*Psmd11*	**105**
	**15D3**	**62283939**	*Pvt1*	**211**	Myc**	**463**		-		-	
	**15F2**	**95582212**	*Dbx2*	**100**	*Nell2*	**225**	-	-		*Tmem16f*	**36**
	**16B3**	**33700976**	*Heg1*	**13**	*Slc12a8*	**118**	-	*Muc13*	**12**	Itgb5 **	**48**
**Mk2**											
	**1 E4**	**132543823**	*Pfkb2*	**0.4**	*C4bp*	**54**	-	*Yod1*	**0.8**	*AA986890*	**1**
	**6A3**	**25759728**	*Pot 1a*	**14**	*Grp37*	**120**	-	-		-	
	**8 E1**	**123703449**	*Irf8*	**81**	*Cox4i1*	**149**	-	Foxf1a**	**269**	*Mthfsd*	**280**
	**12C1**	**52786246**	*Hectd1*	**120**	*EG544864*	**35**	-	*Heatr5a*	**11**	*6530401NO4Rik*	**124**
	**13D2.3**	**116255725**	*Pelo*	**46**	*Itga1*	**33**	-	-		-	
**Mk3**											
	**1C5**		*Glrp1*	**146**	*Spp2*	***230***	-	*Arl14c*	**161**	*Sh3bp4*	**413**
	**1F**	**139621937**	-				-	*Ptprc*	**257**	*Atp6v1g3*	**466**
	**2H3**	**165618991**	Prkcbp1**	**43**	*Eya2*	**156**	-	*Ncoa3*	**65**	*Sulf2*	**146**
	**5B1**	**43941**	*Rheb*	**23**	*Cryng*	**83**	-	Prkag2**	**20**	*1500035N22Rik*	**144**
	**6D2**	**88086902**	*Rpn1*	**16**	*EG434064*	**115**	-	Gata2**	**72**	*Dnajb8*	**100**
	**7B4**	**45356428**	*Bax*	**21**	*Flt1*	**28**	*Dhdh*	*Nucb1*	**4**	*Tulp2*	**25**
	**7F5**	**144856850**	Ccnd1**	**107**	*Oraov1*	**167**	-	*Tpcn2)*	**167**	*Mrgprf*	**253**
	**13A1**	**3830652**	*Calm13*	**27**			**-**	*Calm4*	**6**	*Calm5*	***22***
	**14C3**	**53491694**	*Prmt5*	**20**	*Rem2*	**57**	**-**	*D14Ertd500e*	**4**	*Jub*	**31**
	**19A**	**4301566**	*Ankrd13d*	**18**	*Ssh3*	**32**	Adrbk1**	*Fbxl13*	**15**	*FBXL13*	**15**

* the 2 most proximal genes located at a maximum distance of 500kb upstream (5') or downstream (3') of the integration site

** genes present in the RTCGD database

## Discussion

### Characterization of genes specific to erythro- and megakaryoblastic leukaemias

Patient survey studies revealed that erythroleukaemias represent an average of 5% of all cases of acute myeloid leukaemias [3-5] and megakaryoblastic leukaemias have an incidence of approximately 1% in adults and 5-10% in children [6,7]. However, the overall survival rate is extremely poor and ranges from 6% to 17% [3,7]. Children suffering from Down Syndrome are an exception as they have a higher risk of developing megakaryoblastic leukaemias but respond better to therapy [54]. Acute erythro- and megakaryoblastic leukaemias are less studied than the more frequent types of leukaemias. Thus, genes involved in the development of these leukaemias remain insufficiently elucidated.

Our experimental design is based on the comparison of non-lymphoid versus lymphoid murine leukaemias and provides the whole picture of genes specific to these 2 groups and subgroups. The sole comparison of the non-lymphoid leukaemias and their respective controls without including the lymphoid group would not have provided such a dataset. Therefore, numerous genes not described previously or uncharacterized emerged from this study. We estimated that, within the identified gene signatures, there are oncogenes directly implicated in the disease and also genes related to the normal commitment of the cells toward the erythro- or megakaryoblastic lineages. To determine which genes are potential oncogenes, we first compared the erythroleukaemias to the erythroid control samples and to the study of Welch and colleagues [40]. The comparison with the Welch's study enabled to assess the differentiation state of the leukaemias and the control. Consequently, we could assume that the genes under-expressed in comparison to the control are late stage genes and that oncogenes are more likely to be within the over-expressed genes. We validated this hypothesis with Ltbp2 and Btbd14a in a differentiation assay in the HB22.2 erythroid cell line. Second, to gain more insights into the function of the megakaryoblastic genes and in the absence of a control sample, we compared our list of genes to the study of Shivdasani and colleagues [37]. The comparison with the studies of Welch for the erythroid leukaemias [40] and of Shivadasani [37] for the megakaryoblastic leukaemias provides valuable information about the behaviour of the genes during normal differentiation. However, their respective microarray chips contained less probesets than ours and we could not perform the comparison on the whole dataset. Therefore, further experiments are required to identify the unknown role played by these genes in erythro- and megakaryoblastic leukaemias.

### Erythroid and megakaryoblastic genes

Erythroid and megakaryoblastic lineages, emerging from the same bipotent progenitor, are very closely related [11] and, as confirmed by our study, several transcription factors are commonly expressed. This strengthens the hypothesis that a very fine tuning of these factors influences the commitment toward the erythroid or megakaryocytic lineages.

Our microarray data indicate that *Gata1*, *Gata2*, *Fog1*, *Scl *and *Lmo2 *are expressed both in the Graffi-induced erythro- and megakaryoblastic leukaemias (Table 1). They are known to act on the promoter of their target as multimeric complexes. Our study highlights that *PU.1 *(*Sfpi1*), *Ctbp2*, *Cbfa2t3h *(*Eto2*), *Evi1 *and *Runx1 *have a strong megakaryoblastic pattern. *PU.1 *is a known determinant of erythroid versus megakaryoblastic differentiation and the Gata2 protein acts on PU.1 [55]. The Cbfa2t3h protein binds to the multimeric complex formed by Gata1, Fog1, Scl and Lmo2 and is known to repress the transcription of the target genes. The co-repressor Ctbp2 is known to bind to Evi1 and Fog1 [56]. Runx1 cooperates with Gata1 during megakaryocytic commitment [22,23] and the Runx1-Evi1 fusion protein leads preferentially to the development of megakaryoblastic leukaemias in transgenic mice [57]. In a model of *in vitro *differentiation, *Evi1 *is strongly induced and sustained upon thrombopoietin treatment of CD34^+ ^cells in a pattern very similar to *Gata2 *and *PU.1 *but only weakly upon erythropoietin treatment [58]. Great evidence indicates that Evi1 is a direct activating target of Gata2 [59]. Thus, our study reinforces the importance of these genes in the megakaryoblastic leukaemias.

### RT-PCR validated megakaryoblastic genes

The specific megakaryoblastic expression of several genes with poorly elucidated physiological roles was validated by RT-PCR. Our study reports for the first time *Gucy1a3*, *Gulp1 *and *Fkbp9 *as being specific to megakaryoblastic leukaemias. The function of these genes, related to the normal development or transformation of megakaryocytic cells, has yet to be elucidated. Insight into their physiological roles can be provided by their already known functions in other cell types. *Gucy1a3 *is known to heterodimerize with *Gucy1b3*, which gene is also specific to the Graffi-induced megakaryoblastic leukaemias. The *Gucy1a3/b3 *complex produces cGMP after activation by nitric oxide (NO) itself produced by the NADPH oxidase from reactive oxygen species. As expected, *Ncf2 *and other components of the NADPH oxidase (*Ncf1*, *Ncf4*, *Cybb*) are specifically over-expressed in the Graffi-induced megakaryoblastic leukaemias (Figure 2 and not shown). In human cancerous glioma cell lines, it is hypothesized that *GUCY1a3/b3 *may be responsible for *VEGF *over-expression resulting in an increased amount of NO [60]. NO is also known to play a role in platelet activation [61]. *Gulp1 *could be involved in the intracellular vesicular trafficking [62] which is of high importance in megakaryocytes for transporting the molecules in the storage organelles and during proplatelet formation. *Fkbp9 *is poorly studied and this present study reports its expression for the first time in cells of haematopoietic origin. It is strongly expressed in our megakaryoblastic leukaemias, in human non-lymphoid leukaemias HEL, K562, CMK, Meg-01 and LAMA84, and to a lesser extent, in the murine erythroid leukaemias and cell line.

### RT-PCR validated erythroid genes

The selected erythroid genes with poorly elucidated physiological roles were *Slamf1*, *Snca*, *Ltbp2*, *Rabgef1*, *Cda *and *Btbd1a*. *Slamf1 *is known to be expressed by activated lymphocytes but not yet identified in relation to erythroid leukaemias. The expression of *Slamf1 *in Friend virus-induced erythroleukaemic cell line HB22.2 confirms the Graffi model. The gene was recently revealed as a marker of haematopoietic stem cells distinguishing these cells from more differentiated progenitors [63]. *Snca *has already been reported in erythrocytes [64,65]. Its over-expression in the control sample and its increased expression during erythroid differentiation (G1E dataset, Table 3) indicate that it may be implicated in normal erythroid cells function. We did not observe a significant increase during HB22.2 induced differentiation. *Ltbp2 *is strongly expressed in our erythroleukaemias and increases significantly during differentiation. It shows a non-lymphoid expression pattern in the tested human cell lines and is identified in relation to haematopoietic cells for the first time. Some studies suggest a role for *Ltbp2 *in cell adhesion and in cell migration [66]. *Rabgef1 *has never been reported in relation to erythroid lineage or leukaemia and the encoded protein is known to interact with Rab5, Rab21 or Rab22 [67]. *Rab22a *is indeed specifically over-expressed in the 3 tested erythroleukaemias (not shown). RAB proteins are implicated in the intra-cellular vesicular traffic regulation and *Rabgef1 *is expressed in mast cells where it acts on Kit internalization [68]. Even though *Rabgef1 *expression pattern is more erythroid, it was amplified in all other Graffi-induced leukaemias and in all the tested human cell lines, indicating its ubiquitous expression in haematopoietic cells. *Cda*, responsible for resistance to chemotherapy treatment, is highly expressed in our erythroleukaemias, in HB22.2, K562 and HEL. The GEO database shows that *CDA *is up-regulated during the differentiation of human CD34^+ ^cells toward the erythroid lineage (NCBI GEO, GSE4655). As of this day, *Btbd14a *has never been studied. It is highly expressed in the Graffi-induced erythroleukaemias and in HB22.2. Finally, *Btbd14a *appears ubiquitously expressed in the haematopoietic system. The BTB/POZ domain of *Btb14a *is present in many oncogenes involved in the development of leukaemia and is often found at the N-terminus of transcription factors. Thus, this gene is particularly interesting to further study as it shows a decreased expression during HB22.2 induced differentiation and could represent a potential oncogene.

### The MkMB Signature

Numerous genes were commonly over-expressed in the megakaryoblastic, the myeloid leukaemias and/or the B-cell leukaemias. These genes are, in most cases, already known to be expressed by cells implicated in innate immunity. Platelets function is too often considered limited to blood coagulation and formation of thrombosis but some studies now emphasize that the platelets' role is underestimated in innate immunity and inflammation response [39,69-72]. Upon activation, platelets can release microbicidal proteins, interleukins that trigger a general inflammation response and chemokines that recruit immune cells such as leukocytes. Pathogens such as bacteria and lentiviruses can be ingested by platelets [73]. The MkMB signature seems to reflect partly the complexity of the platelet function. Only very few examples are shown in Table 2 but the complete list is available in the supplementary data [19]. The unique design of this gene expression study that compared different types of leukaemias highlights this signature.

### Retroviral integration and genes potentially implicated in the onset of the disease

The analysis of retroviral integration enables the identification of genes that may be responsible for malignant transformation. In this study, we screened the 3 megakaryoblastic leukaemias for viral integration sites as oncogenic transformation events leading to megakaryoblastic leukaemias remain unknown. No CIS were identified but some genes in the RTCGD have drawn our attention due to their known functions. Within these genes, *Kit *and *Gata2 *are of particular interest as accumulating evidences point at their role in megakaryopoiesis and megakaryoblastic leukaemias.

*Gata2 *was recently reported for the first time as a common integration site in leukaemias induced by the MOL4070LTR retrovirus in the NHD13 mouse [74]. *In vitro *studies showed that *Gata2 *over-expression redirects the haematopoietic differentiation from the macrophage lineage toward the erythroid or the megakaryocytic lineages or from the erythroid toward the megakaryocytic lineage [55,75]. The importance of *Gata2 *on megakaryopoiesis was also demonstrated in a differentiation study in which this gene is strongly induced and sustained upon thrombopoietin treatment of CD34^+ ^cells but only weakly induced upon erythropoietin treatment [58]. The authors made the interesting hypothesis that *GATA2 *might repress the expression of the erythroid markers in maturing megakaryocytic cells since its activation inhibits erythroid differentiation in some systems.

*Kit *is involved in many cancers and is regulated by the SCL complex (*Gata1/2*, *SCL*, *Lmo2*) in haematopoietic cells [76]. More evidence begins to emerge for its role in very early stages of megakaryopoiesis [77,78] and in megakaryoblastic leukaemias [79,80]. Bourquin *et al. *reported increased levels of *KIT*, *GATA2 *and *MYC *in DS AMKL cells harbouring a *GATA1 *mutation compared to non-DS AMKL cells. They hypothesized that the mutated *GATA1 *in DS AMKL cells failed to repress the expression of these 3 genes [79].

The 3 megakaryoblastic leukaemias express very high levels of *Kit *and *Gata2*. We hypothesize that the viral integrations may block the repression of these genes by continuously activating the transcription or inhibiting the binding of repressor molecules. *Kit *and *Gata2 *levels normally decrease during megakaryocytic differentiation (Table 2 'GSE6593'). These 2 genes, affected by the retroviral integration, would send continuous signals of proliferation and survival to the cell. The analysis of a larger sample of Graffi-induced megakaryoblastic leukaemias would be required to prove more efficiently the involvement of *Gata*2 and *Kit *in this particular type of leukaemia.

## Conclusions

In this report, we compared the gene profiles of the erythro- and megakaryoblastic leukaemias induced by the Graffi virus. Our study identifies genes that are highly expressed in the Graffi-induced erythro- and megakaryoblastic leukaemias. The complete dataset of this study is readily available (GSE12581 and [19]). Within the data, numerous genes have not yet been assigned with a known function and some of them could be used as markers for specific types of leukaemias and even the target of new therapies. We selected and RT-PCR validated genes those functions in normal cells are poorly characterized. For the majority, their expression in these lineages is shown for the first time and further functional characterization will surely complement the knowledge of erythroid and megakaryocytic lineages.

## Competing interests

The authors declare that they have no competing interests.

## Authors' contributions

VV designed and performed the microarray, RT-PCR, differentiation assay and cloning of the viral insertion sites experiments, analyzed and interpreted the data, and wrote the manuscript. PL performed the RT-PCR experiments on the human haematopoietic cell lines. DPSO optimized the protocol for the cloning of viral integration sites. YBD provided the HB22.2, K562 and HEL cell lines and critically revised the manuscript. ER guided the project and wrote the manuscript. All authors read and approved the final manuscript.

## Pre-publication history

The pre-publication history for this paper can be accessed here:

http://www.biomedcentral.com/1755-8794/3/2/prepub

## Supplementary Material

Additional file 1 **Oligonucleotides utilized in the RT-PCR experiments**. sequences of forward and reverse primers utilized in the RT-PCR experiments Click here for file

Additional file 2 **Immunophenotype of the leukaemic samples selected for the microarray experiments**. the table lists the leukaemias included in the microarray experiments including sample name, leukaemia type, immunophenotype, antibody used for sorting and tumor origins. Click here for file
