# Supplementary material for: Gene profiling of the erythro- and megakaryoblastic leukaemias induced by the Graffi murine retrovirus
Source: BMC Med Genomics. 2010 Jan 26;3:2. doi: 10.1186/1755-8794-3-2 (PMC2843641; doi:10.1186/1755-8794-3-2)
Supplement: Additional file 1 — Oligonucleotides utilized in the RT-PCR experiments. sequences of forward and reverse primers utilized in the RT-PCR experiments [file 1755-8794-3-2-S1.DOC]

| **Additional File 1 - Oligonucleotides utilized in the RT-PCR experiments** | | |
| --- | --- | --- |
|  | **forward oligo (5'3')** | **reverse oligo (5'3')** |
| **Murine genes** |  |  |
| ***Gata2*** | **TTGTACGTTGCCACAGAAGC** | **TCGAAGCTTGGGACACTTTT** |
| ***Kit*** | **TTATCCTTTAGGCCGTGTGG** | **TGTGGCCCCTTAAGTACCTG** |
| ***Ncf2*** | **TCTAAGAAGCTGGCGCTCTC** | **GCGTCTGAGTTTTCCCTTTG** |
| ***Gucy1a3*** | **AGGCCTTGCGAAGGTCTATT** | **CTTCACACCGCTTGGGTTAG** |
| ***Fkbp9*** | **CACAGACCAAATGGAAGCTG** | **GACCTCAAATTGCCTTCCAA** |
| ***Gulp1*** | **TTCCACAACATTTGCCCATA** | **CCATAGGGCATCATCATTCA** |
| ***Slamf1*** | **TCTCTCCGTTGGTTCTGAGG** | **TGCCAAAGTTGTGCAAGAAG** |
| ***Snca*** | **GATCCTGGCAGTGAGGCTTA** | **CAGCACACAAGACCCTGCTA** |
| ***Ltpb2*** | **CCAGCCTTGCCAAATTTTTA** | **AGCCACTGTCCCCATTACAG** |
| ***Rabgef1*** | **TTATTTGGGGCTGGTTTCTG** | **GGGAAATCAGGCAGGACATA** |
| ***Cda*** | **AAAACGCCTGCTACCCACTA** | **TCCTGGACCGTCCTGACTAC** |
| ***Btbd14a*** | **CTGAGAACTGATGCGGTGAA** | **ACAAGGTCCCTGCATAGGTG** |
| ***Hba*** | **AAGCCCTGGAAAGGATGTTT** | **GGCTCAGGAGCTTGAAGTTG** |
| ***Gapdh*** | **TGTTCCTACCCCCAATGTGT** | **CCCTGTTGCTGTAGCCGTAT** |
| **b-actin** | **GACGGCCAGGTCATCACTAT** | **GTACTTGCGCTCAGGAGGAG** |
| **Human genes** |  |  |
| ***NCF2*** | **GACCAAGGCTTTCCAGATGA** | **GTGCCCTTTCCAGACACTTC** |
| ***GUCY1A3*** | **TCAAGATGCGAATTGGACTG** | **TCCAGAAAATGGCAGATTCC** |
| ***FKBP9*** | **TGCCCAGCCACTTAGTTTTT** | **CAGGTGGACTGTCATTGCTG** |
| ***GULP1*** | **TGAGCCTTCTTGTTTAGTTTCTTTT** | **TCATGCCAGTAGCAGAGTTGA** |
| ***SLAMF1*** | **GGACAGACCCCTCAGAAACA** | **TGTTGGTCTCTGGTGTCAGC** |
| ***SNCA*** | **CCACAGGAAGGAATTCTGGA** | **CCACAAAATCCACAGCACAC** |
| ***LTBP2*** | **GCAGCAAGAGGACATGATGA** | **TTGGAAATCTGGCTGCTTCT** |
| ***RABGEF1*** | **CAGGCAGCTGAGAAAGGTTC** | **CCAAAGAAGGGCAAGTTTTG** |
| ***CDA*** | **GCCGTCTCAGAAGGGTACAA** | **ACATCATCTTTGCCCAGTCC** |
| ***BTBD14A*** | **GGTCCTGAACGCTGTGAAAT** | **GGCACTCAGGTCAACATTCA** |
| ***GAPDH*** | **GTCAGTGGTGGACCTGACCT** | **CCCTGTTGCTGTAGCCAAAT** |
