# Supplementary material for: Gene profiling of the erythro- and megakaryoblastic leukaemias induced by the Graffi murine retrovirus
Source: BMC Med Genomics. 2010 Jan 26;3:2. doi: 10.1186/1755-8794-3-2 (PMC2843641; doi:10.1186/1755-8794-3-2)
Supplement: Additional file 2 — Immunophenotype of the leukaemic samples selected for the microarray experiments. the table lists the leukaemias included in the microarray experiments including sample name, leukaemia type, immunophenotype, antibody used for sorting and tumor origins. [file 1755-8794-3-2-S2.DOC]

| **Additional File 2 - Immunophenotype of the leukaemic samples selected for the microarray experiment** | | | | |
| --- | --- | --- | --- | --- |
| **Leukaemia** | **Sample Name** | **Phenotype** | **Sorting*** | **Organs** |
| **T-cell** | **T1** | **CD4^+^CD8^+^** | **CD4** | **thymus** |
| **T-cell** | **T2** | **CD4^-^CD8^+^** | **CD4** | **thymus** |
| **T-cell** | **T3** | **CD4^+^CD8^-^** | **CD8** | **thymus** |
| **B-cell** | **B1** | **B220^+^CD19^+^** | **CD19** | **lymph nodes** |
| **B-cell** | **B2** | **B220^+^CD19^+^** | **CD19** | **lymph nodes** |
| **B-cell** | **B3** | **B220^low^CD19^+^Sca-1^+^** | **CD19** | **lymph nodes** |
| **Myeloid** | **M** | **CD11b^+^Gr-1^+^** | **CD11b** | **spleen** |
| **Erythroid** | **E1** | **Ter119^+^CD71^+^** | **Ter119** | **spleen***** |
| **Erythroid** | **E2** | **Ter119^+^CD71^+^** | **Ter119** | **spleen***** |
| **Erythroid** | **E3** | **Ter119^+^CD71^+^** | **Ter119** | **spleen***** |
| **Megakaryoblastic** | **Mk1** | **cKit^+^CD41^-^** | **Kit** | **spleen** |
| **Megakaryoblastic** | **Mk2** | **cKit^+^CD41^+^** | **CD41** | **spleen** |
| **Megakaryoblastic** | **Mk3** | **cKit^+^CD41^+^** | **CD41** | **spleen** |
| **Control samples:** | | | | |
| **T-cell control** | **CT** | **CD4^+^CD8^+ (**)^** | **CD4, CD8** | **thymus** |
| **B-cell control** | **CB** | **B220^+^CD19^+ (**)^** | **CD19** | **bone marow** |
| **Erythroblasts** | **CE** | **Ter119^+^CD71^+(**)^** | **Ter119** | **bone marrow***** |
| *** antibodies used for cell sorting, **n=12; *** red blood cell lysis** | | | | |
